# Interim estimates of 2019/20 vaccine effectiveness during early-season co-circulation of influenza A and B viruses, Canada, February 2020

**DOI:** 10.2807/1560-7917.ES.2020.25.7.2000103

**Published:** 2020-02-20

**Authors:** Danuta M Skowronski, Macy Zou, Suzana Sabaiduc, Michelle Murti, Romy Olsha, James A Dickinson, Jonathan B Gubbay, Matthew A Croxen, Hugues Charest, Agatha Jassem, Mel Krajden, Nathalie Bastien, Yan Li, Gaston De Serres

**Affiliations:** 1 British Columbia Centre for Disease Control, Vancouver, Canada; 2 University of British Columbia, Vancouver, Canada; 3 Public Health Ontario, Toronto, Canada; 4 University of Toronto, Toronto, Canada; 5 University of Calgary, Calgary, Canada; 6 Public Health Laboratory (ProvLab), Alberta Precision Laboratories, Edmonton, Alberta, Canada; 7 University of Alberta, Edmonton, Canada; 8 Institut National de Santé Publique du Québec, Québec, Canada; 9 National Microbiology Laboratory, Public Health Agency of Canada, Winnipeg, Canada; 10 Laval University, Quebec, Canada; 11 Centre Hospitalier Universitaire de Québec, Québec, Canada

**Keywords:** influenza, vaccine effectiveness, genetic sequencing, clade, antigenic match

## Abstract

Interim results from Canada's Sentinel Practitioner Surveillance Network show that during a season characterised by early co-circulation of influenza A and B viruses, the 2019/20 influenza vaccine has provided substantial protection against medically-attended influenza illness. Adjusted VE overall was 58% (95% confidence interval (CI): 47 to 66): 44% (95% CI: 26 to 58) for A(H1N1)pdm09, 62% (95% CI: 37 to 77) for A(H3N2) and 69% (95% CI: 57 to 77) for influenza B viruses, predominantly B/Victoria lineage.

The 2019/20 northern hemisphere influenza season has been characterised by early co-circulation of influenza A and B viruses [1-5]. We report interim virological and vaccine effectiveness (VE) findings for the 2019/20 season from the community-based Canadian Sentinel Practitioner Surveillance Network (SPSN).

## Study design

VE was estimated using a test-negative design as previously described [6]. Nasal/nasopharyngeal specimens were collected from patients presenting to sentinel sites in the provinces of Alberta, British Columbia, Ontario and Quebec. Patients who were at least 1 year of age and who presented within 7 days of onset of influenza-like illness (ILI) were eligible for inclusion in VE analyses. ILI was defined by self-reported fever and cough and one or more of arthralgia, myalgia, prostration or sore throat. Fever was not a requirement for adults aged ≥ 65 years old. Influenza vaccination status was based on self- (or parent/guardian) report of 2019/20 vaccine receipt ≥ 2 weeks before ILI onset.

Specimens were tested for presence of influenza virus by real-time RT-PCR assays. Sanger sequencing of the haemagglutinin (HA) gene was undertaken on a convenience sample of original patient specimens. Amino acid substitutions at HA antigenic sites are hereafter specified in parentheses, those affecting the receptor-binding site as ‘RBS’ and changes associated with potential gain or loss of N-linked glycosylation as ‘+/−CHO’. Viral sequence data were deposited for reference into the Global Initiative on Sharing All Influenza Data (GISAID) platform (www.gisaid.org) under accession numbers EPI_ISL_41122–411846. Antigenic characterisation of a convenience sample of virus isolates was undertaken by haemagglutination inhibition (HI) assay using post-infection ferret anti-sera raised to egg-passaged influenza A and cell-passaged influenza B vaccine reference strains, conducted as previously described [6-8].

Adjusted odds ratios (OR) for influenza test-positivity between vaccinated and unvaccinated participants were derived using a logistic regression model. VE was calculated as (1 − adjusted OR) × 100%.

## Ethical statement

The 2019/20 VE study protocol was approved by ethics review committees: University of Calgary, Calgary, Alberta (REB15–0587_MOD9); University of Alberta, Edmonton, Alberta (Pro00097554); University of British Columbia, Vancouver, British Columbia (H04–80634); Public Health Ontario, Toronto, Ontario (2017–057.03); and Comité d’éthique de santé publique, Québec.

## Influenza vaccine components and formulations

For the 2019/20 influenza vaccine, the World Health Organization recommended update to both influenza A vaccine components from the prior 2018/19 season, changing from clade 6B.1 to a clade 6B.1A1 strain for A(H1N1)pdm09 (A/Brisbane/02/2018-like); and from clade 3C.2a1 to a clade 3C.3a strain for A(H3N2) (A/Kansas/14/2017-like) [9,10]. The influenza B vaccine components were unchanged from the prior season: trivalent vaccine included a B/Victoria-lineage clade V1A.1 (Δ2) strain (B/Colorado/06/2017-like) defined by a double amino-acid deletion in the 160-loop of the HA protein; quadrivalent influenza vaccine additionally included a clade 3 B/Yamagata-lineage virus (B/Phuket/3073/2013-like) [9,10].

All influenza vaccines used in Canada were manufactured in eggs and inactivated. Overall and by province ≥ 74% of publicly-funded doses were quadrivalent except in British Columbia where 16% of doses overall were quadrivalent and targeted to children. In Ontario high-dose trivalent vaccine was publicly funded for elderly adults aged ≥ 65 years old.

## Study period and influenza detection

The study period spanned specimen collection dates from 1 November 2019 (week 44) to 1 February 2020 (week 5) during which 2,808 specimens met inclusion criteria. Of these, 1,411 (50%) were influenza test-positive including 731 (52%) influenza A and 683 (48%) influenza B viruses, with three influenza A and B co-infections. Of the 715 influenza A cases of known subtype, 551 (77%) were A(H1N1)pdm09 and 164 (23%) were A(H3N2). Among the 683 influenza B detections, lineage was known for 262 (38%), of which 261 (99%) were B/Victoria (Figure).

**Figure:**
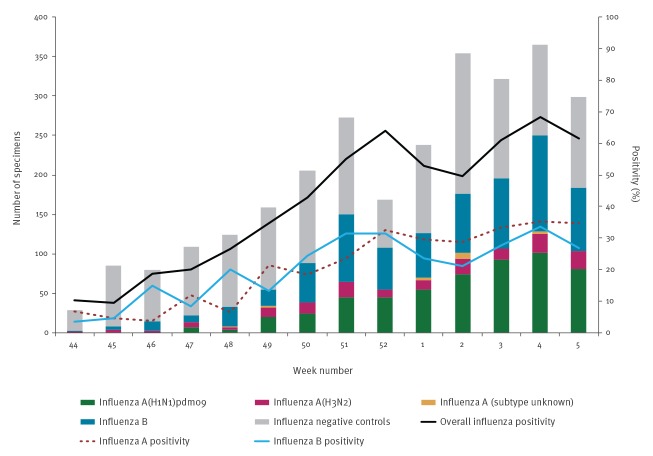
Influenza detections among specimens collected from eligible patients presenting with influenza-like illness, by week of specimen collection, Canadian Sentinel Practitioner Surveillance Network, 1 November 2019–1 February 2020 (n = 2,811^a^)

^a^ The epidemic curve includes three influenza A and B co-infections, two involving influenza A(H1N1)pdm09 plus influenza B and one involving influenza A of unknown subtype plus influenza B. These six viruses from three specimens are each plotted separately giving 2,811 displayed specimens rather than 2,808.

Missing specimen collection dates were imputed as the date the specimen was received and processed at the provincial laboratory minus 2 days.

## Participant characteristics

As in prior seasons [6,8], most (61%; 1,718/2,808) participants were adults 20–64 years old (Table 1). Among test-negative controls, 21% (295/1,397) had one or more comorbidities, which is comparable to last season's interim report (22%) and consistent with other surveillance data indicating > 20% of Canadians live with a major chronic disease [11]. Vaccination ≥ 2 weeks before ILI onset was reported by 29% (399/1,397) of controls overall and 26% (229/877) of those 20–64 years old, also similar to last season's interim report (27% and 24%, respectively) [8].

**Table 1:** Interim vaccine effectiveness (VE) estimates against influenza, Canadian Sentinel Practitioner Surveillance Network (SPSN), 1 November 2019–1 February 2020 (n = 2,808)

Influenza outcome	Age group (years)	Total	Cases			Controls			Adjusted VE %^a,b,c^	95% CI
			All	Vaccinated	%	All	Vaccinated	%		
Any A or B^d^	All ages	2,808	1,411	191	14	1,397	399	29	58	47 to 66
	1–19	866	512	33	6	354	70	20	74	59 to 84
	20–64	1,718	841	122	15	877	229	26	55	41 to 66
	≥65^e^	224	58	36	62	166	100	60	18	−59 to 58
Influenza A	All ages	2,128	731	131	18	1,397	399	29	49	34 to 60
	1–19	543	189	15	8	354	70	20	70	44 to 84
	20–64	1,372	495	88	18	877	229	26	45	25 to 59
	≥65	213	47	28	ND	166	100	60	NE	
A(H1N1)pdm09	All ages	1,948	551	107	19	1,397	399	29	44	26 to 58
	1–19	478	124	13	10	354	70	20	63	25 to 81
	20–64	1,273	396	75	19	877	229	26	39	14 to 56
	≥65	197	31	19	ND	166	100	60	NE	
A(H3N2)	All ages	1,561	164	22	13	1,397	399	29	62	37 to 77
	1–19	414	60	2	3	354	70	20	NE	
	20–64	967	90	11	12	877	229	26	64	29 to 81
	≥65	180	14	9	ND	166	100	60	NE	
Influenza B^f^	All ages	2,080	683	60	9	1,397	399	29	69	57 to 77
	1–19	679	325	18	6	354	70	20	77	59 to 87
	20–64	1,224	347	34	10	877	229	26	68	51 to 79
	≥65	177	11	8	ND	166	100	60	NE	

CI: confidence interval; ND: not displayed owing to small denominator; NE: not estimated owing to sparse data; VE: vaccine effectiveness.

^a^ All VE estimates adjusted for age group, province (Alberta, British Columbia, Ontario, Quebec), specimen collection interval (≤ 4; 5–7 days) and calendar time (modelled as a natural cubic spline with three equally-spaced knots). For all ages, age group adjustment based on 1–8, 9–19, 20–49, 50–64 and ≥ 65 years. For children 1–19 years old, age adjustment based on 1–8 and 9–19 years. For adults 20–64 years old, age adjustment based on 20–49 and 50–64 years.

^b^ Additional adjustment for comorbidity (yes/no/unknown) and sex (male/female/unknown) did not alter any of the displayed VE estimates by more than 2% (absolute) except where specified.

^c^ Using a later study start date of 1 December 2019 did not alter any of the displayed VE estimates by more than 3% (absolute).

^d^ Excluding the province of British Columbia where a smaller proportion of doses distributed were quadrivalent, the VE estimate for all ages was unchanged and age-stratified estimates remained within 6% (absolute) of those displayed.

^e^ With additional adjustment for comorbidity (yes/no/unknown) and sex (male/female/unknown), VE was 14% (95% CI: −71 to 57).

^f^ Excluding the province of British Columbia, none of the influenza B VE estimates were higher and all remained within 5% (absolute) of those displayed.

## Vaccine effectiveness and virological characterisation

The 2019/20 influenza VE overall was 58% (95% CI: 47 to 66), reflecting the preponderance of contributing adults 20–64 years old (55%; 95% CI: 41 to 66), with higher point estimates among children 1–19 years (74%; 95% CI: 59 to 84) but lower among adults aged ≥65 years (18%; 95% CI: −59 to 58) (Table 1).

### Influenza A(H1N1)pdm09

VE against influenza A(H1N1)pdm09 was 44% (95% CI: 26 to 58) overall: 63% (95% CI: 25 to 81) in children 1–19 years old and 39% (95% CI: 14 to 56) in adults 20–64 years old (Table 1). Of the 551 influenza A(H1N1)pdm09 viruses detected by the SPSN and contributing to VE analyses, 287 (52%) were sequenced. This showed that none of the A(H1N1)pdm09 viruses belonged to the same clade as the vaccine strain (6B.1A1). Instead, 285/287 (99%) viruses belonged to clade 6B.1A5 of which 245 (86%) further sub-clustered with 6B.1A5A and 39 (14%) with 6B.1A5B (Table 2).

**Table 2:** Clade distribution of viruses contributing to influenza vaccine effectiveness (VE) analyses, Canadian Sentinel Practitioner Surveillance Network (SPSN), 1 November 2019–1 February 2020 (n = 628)

Clades with defining substitutions (antigenic site) + extra substitutions (antigenic site)	**Number of viruses**
**Influenza A(H1N1)pdm09**	**N = 287**
**6B.1A = 6B + S74R (Cb) + S162N (Sa)(+CHO) + S164T (Sa) + I216T + I295V**	**n = 0**
**6B.1A1^a^= 6B.1A + S183P**	**n = 0**
**6B.1A5 = 6B.1A + S183P + N260D**	**n = 1**
**6B.1A5A = 6B.1A5 + N129D + T185I (Sb)**	**n = 245**
+ D187A (Sb)(RBS) + Q189E (Sb)	99
+ D187A (Sb)(RBS) + Q189E (Sb) + A73E (Cb) + T120I	16
+ K130N + N156K (Sa) + L161I (Sa) + V250A + HA2: T147A	108
**6B.1A5B = 6B.1A5 + E235D (Ca1) + HA2: V193A**	**n = 39**
+ K160M (Sa) + T216K	2
+ K160M (Sa) + T216K + K130N + H296N	15
+ K160M (Sa) + T216K + K130N + H296N + P137S (Ca2) + V272I	22
**6B.1A7 = 6B.1A + K302T + HA2: I77M + N169S + E179D**	**n = 2**
+ E68D + S121N + L161I (Sa) + T120A	1
**Influenza A(H3N2)**	**N = 80**
**3C.2a1b = 3C.2a^b^ + N171K (D)** + **N121K (D) + K92R (E) + H311Q (C) + HA2: I77V + G155E**	**n = 0**
**3C.2a1b/T131K = 3C.2a1b + E62G (E) + R142G (A) + T131K (A) + HA2:V200I**	**n = 44**
+ K83E (E) + Y94N (E)	4
+ Q197R (B) + S219F (D) + HA2: V18M	3
+ Q197R (B) + S219F (D) + HA2: V18M + K207R (D)	32
+ Q197R (B) + S219F (D) + HA2: V18M + K207R (D) + S144R (A)	4
**3C.2a1b/T135K = 3C.2a1b + E62G (E) + R142G (A) + T135K (A)(RBS)(−CHO) + T128A (B)(−CHO)**	**n = 31**
+ S137F (A)(RBS) + A138S (A)(RBS) + F193S (B)	2
+ S137F (A)(RBS) + A138S (A)(RBS) + F193S (B) + E50K (C)	8
+ A138S (A)(RBS) + G186D (B) + D190N (B)(RBS) + F193S (B) + S198P (B)	19
**3C.3a^a^ = 3C.3^c^ + L3I + S91N (E) + A138S (A)(RBS) + N144K (A)(−CHO) + F159S (B) + F193S (B) + N225D (RBS) + K326R + HA2: D160N**	**n = 5**
**Influenza B/Victoria lineage**	**N = 260**
**V1A.1 (Δ2)^a^ = V1A + Δ162–163 (160-loop) + D129G (120-loop) + I180V + HA2: R151K**	**n = 1**
**V1A.3A (Δ3) = V1A + Δ162–164 (160-loop) + I180T + K209N**	**n = 0**
**V1A.3B (Δ3) = V1A + Δ162–164 (160-loop) + K136E (120-loop)**	**n = 259**
+ G133R (120-loop) + E128K (120-loop)	117
+ R133K (120-loop) + E128K (120-loop)	79
+ N150K (150-loop) + G184E + N197D (190-helix)(−CHO) + R279K	1
**Influenza B/Yamagata lineage**	**N = 1**
**Clade 3^d^**	**n = 1**
+ R48K + L172Q + D232N (230-region)( + CHO) + M251V	1

HA: haemagglutinin; (+/ − CHO) signifies gain/loss of potential N-linked glyscosylation; (RBS) signifies substitution affecting the receptor binding site.

The number of viruses belonging to the specified influenza A subtype or B lineage are shown in bolded font as N=number. The number of viruses belonging to a parent genetic group are also shown in bold as n=number. The number of viruses within that parent group bearing the additional substitutions specified are shown in normal font. Specified substitutions are for HA1 unless specified as for HA2.

^a^ Indicates 2019/20 trivalent influenza vaccine strain.

^b^ Clade 3C.2a defined by 3C + L3I + N144S (A) + N145S (A) + F159Y (B) + K160T (B) (+ CHO) + N225D (RBS) + Q311H (C) + HA2: D160N.

^c^ Clade 3C.3 defined by 3C + T128A(B)(−CHO) + R142G (A) + N145S(A).

^d^ Indicates 2019/20 quadrivalent influenza vaccine strain. SPSN virus bears additional substitutions in relation to the vaccine strain as shown.

With restriction to the 245 influenza A(H1N1)pdm09 cases belonging to clade 6B.1A5A, the VE was 49% (95% CI: 26 to 65). Among the 6B.1A5A viruses, two distinct genetic sub-groups were observed. This includes 115 (47%) viruses that bore additional antigenic site Sb substitutions, namely D187A (Sb)(RBS) and Q189E (Sb), and for which VE was 61% (95% CI: 30 to 78). The second sub-group includes 108 (44%) viruses that instead bore new antigenic site Sa substitutions, namely N156K (Sa) and L161I (Sa), for which VE was 45% (95% CI: 6 to 68). All 39 of the 6B.1A5B viruses also showed drift, acquiring K160M (Sa) and some also P137S (Ca2) substitution (Table 2). VE against clade 6B.1A5B viruses was 26% (95% CI: −69 to 67). However, these clade-specific analyses are based on limited convenience subsets of the A(H1N1)pdm09 cases, requiring cautious interpretation in this interim analysis. 

Of 87/551 (16%) A(H1N1)pdm09 viruses characterised by HI assay, 41 (47%) were antigenically distinct from the vaccine strain. Sequence information was available for 39/41 and all belonged to the 6B.1A5A sub-group bearing the new Sa substitutions.

### Influenza A(H3N2)

VE against influenza A(H3N2) was 62% (95% CI: 37 to 77) overall (Table 1). Of 80/164 (49%) A(H3N2) viruses sequenced, just five clustered with the clade 3C.3a vaccine strain. Most (75/80; 94%) belonged instead to clade 3C.2a1b, including 44/75 (59%) with T131K (A) and 31/75 (41%) with T135K (A)(RBS)(−CHO) substitution. Within the T135K group, two sub-clusters contributed: one that first arose during the 2018/19 season bearing additional substitutions S137F (A)(RBS), A138S (A)(RBS) and F193S (B) and another newly arisen in 2019/20 bearing A138S (A)(RBS), G186D (B), D190N (B)(RBS), F193S (B) and S198P (B) substitutions. A138S and F193S are parallel substitutions acquired independently in both 3C.2a1b/T135K sub-clusters and 3C.3a viruses, also present in the 2019/20 clade 3C.3a vaccine strain (Table 2). Eight influenza A(H3N2) viruses were successfully characterised by HI of which seven were antigenically distinct from the vaccine strain.

### Influenza B

VE against influenza B was 69% (95% CI: 57 to 77) (Table 1). Of 683 influenza B viruses detected by the SPSN and contributing to VE analyses, 260 (38%) were characterized as B/Victoria lineage by sequencing (one other virus was characterized as B/Victoria lineage by HI assay). Virtually all (259; 99%) sequenced B/Victoria-lineage viruses belonged to clade V1A.3 (Δ3) characterised by a triple deletion at amino acids 162–164 in the 160-loop. All 259 viruses belonged to the V1A.3B sub-cluster with K136E in the 120-loop with most (196/259; 76%) also bearing other 120-loop substitutions. Just one virus belonged to the same B/Victoria clade as the trivalent vaccine strain (V1A.1 (Δ2)) and one other sequenced virus belonged to the same B/Yamagata-lineage as the quadrivalent vaccine strain (clade 3) (Table 2). Fifty-eight viruses were HI-characterised: all but one was distinct from the cell-passaged vaccine strain.

## Discussion

Interim results from Canada's SPSN indicate that the 2019/20 influenza vaccine has provided substantial protection against medically-attended influenza illness during a season characterised by an approximately equal mix of influenza A and B viruses, a substantial proportion of which were genetically and antigenically mismatched to vaccine.

The mid-season VE of 44% we report for A(H1N1)pdm09 viruses during the 2019/20 season is lower than we reported mid-season in 2018/19 (72%) [8], 2015/16 (64%) [12] or 2013/14 (74%) [13]. The 2019/20 A(H1N1)pm09 vaccine component is a clade 6B.1A1 strain defined by S183P substitution whereas clade 6B.1A5 viruses, notably the 6B.1A5A sub-cluster, have predominated so far in Canada and Europe [3]. In addition to S183P, 6B.1A5A viruses bear T185I (Sb) and about half (47%) additionally bear other antigenic site Sb substitutions (D187A (Sb)(RBS) and Q189E (Sb)), with residue 187 in particular recognised for its potential role in the emergence of escape mutants [14-17]. Nearly half (44%) of 6B.1A5A viruses have instead acquired novel substitutions in antigenic site Sa (N156K (Sa) and L161I (Sa)). This recent accumulation of several substitutions clustered within pivotal antigenic sites Sa and Sb suggests immune selection pressure [14,15]; consistent with that, a substantial proportion of A(H1N1)pdm09 viruses characterised by the SPSN (41/87; 47%) and in Canada overall (89/235; 38%) [1] this season have been antigenically distinct from the vaccine strain.

Almost all influenza B viruses belonged to the B/Victoria lineage which has not otherwise contributed much since the 2015/16 season [1,18]. Children are most affected by influenza B, particularly B/Victoria-lineage viruses [19,20], and this may be evident in the over-representation of children 1–19 years old among unvaccinated influenza B cases (307/623; 49%) compared with controls (284/998; 28%) or with the population of SPSN provinces (20%) overall [21]. Whereas the 2019/20 vaccine is a double deletion V1A.1 (Δ2) strain, virtually all viruses collected and sequenced by the SPSN were instead triple deletion V1A.3B (Δ3) variants, as also noted from Europe [3] and the United States (US) [4]. The majority of B/Victoria-lineage viruses HI-characterised by the SPSN (57/58), and otherwise in Canada (157/173; 91%) [1] have also been antigenically distinct from the vaccine strain. Notwithstanding that vaccine mismatch, we found substantial VE of 69% overall and 77% in children. As previously highlighted, influenza B immuno-epidemiology is complex with cohort effects and cross-lineage interactions that may also play a role in vaccine protection [18,19,22-24].

Most but not all A(H3N2) viruses successfully characterised by HI assay to date in Canada (35/41; 85%) [1] and in Europe (11/17; 65%) [3] have been antigenically distinct from the egg-adapted vaccine strain, and in the US most (39/69; 57%) have also been distinct from the cell-passaged vaccine strain based upon focus reduction assay [4]. In that regard, the VE of 62% we report may be unexpected. Effectiveness of the 2019/20 clade 3C.3a vaccine against predominant 3C.2a1b viruses is higher than observed for the 2018/19 clade 3C.2a1 vaccine against late-season A(H3N2) viruses overall (17%) or in clade-specific analyses against co-circulating 3C.2a1b (27%) or 3C.3a (−32%) viruses [25]. Antibody induced to clade 3C.3a may be more cross-reactive than that of antibody induced to clade 3C.2a [26,27], and recent parallel substitutions shared between 3C.3a and 3C.2a1b/T135K viruses (e.g. A138S, F193S) may further contribute. An immunological cohort effect (i.e. imprint-regulated effect of vaccine; I-ReV) was hypothesised last season to explain the paradoxical negative VE for the 3C.2a1 vaccine against 3C.3a viruses, notably among adults 35-54 years of age [25,28,29]. Whether the I-ReV hypothesis may also apply, but in reverse, to explain this season's protective VE for 3C.3a vaccine against 3C.2a1 viruses requires greater sample size to explore. We highlight that only once previously in the past decade (2011/12) has the SPSN reported an overall VE exceeding 50% for A(H3N2) viruses [6]. As such, and particularly noting the limited sample size of A(H3N2) cases, our interim estimate of 2019/20 A(H3N2) VE requires cautious interpretation pending further end-of-season evaluation.

Limitations of the current analysis include its observational design for which residual bias and confounding cannot be ruled out. Sample size considerations preclude further stratification (e.g. by additional age and/or genetic sub-groups, or prior vaccination history) but will be attempted end-of-season. Our analyses reflect specimens and data collected as at 1 February 2020 but may change towards the end of the ongoing epidemic.

## Conclusions

The 2019/20 VE reported by the Canadian SPSN suggests that, among non-elderly individuals, about six of 10 cases of medically-attended febrile respiratory illness due to influenza might have been prevented by vaccination. Such substantial vaccine protection despite antigenic mismatch, notably to circulating influenza A(H3N2) and B/Victoria viruses, invites exploration of other factors potentially contributing to VE.
